# Quantitative analysis of ciliary beating in primary ciliary dyskinesia: a pilot study

**DOI:** 10.1186/2046-2530-1-S1-O3

**Published:** 2012-11-16

**Authors:** J-F Papon, LB Bassinet, GCP Cariou-Patron, FZL Zerah-Lancner, AMV Vojtek, SB Blanchon, BC Crestani, SA Amselem, A Coste, BH Housset, EE Escudier, BL Louis

**Affiliations:** 1 AP-HP, Hôpital H.-Mondor – A. Chenevier,France; 2 Hôpital intercommunal de Creteil, France; 3 AP-HP, Hôpital Armand-Trousseau, France; 4 AP-HP, Hôpital Bichat-Claude Bernard, France; 5 INSERM, U933, France; 6 INSERM, U955, France; 7 Universite Paris Est, Faculté de Médecine, France; 8 CNRS, ERL 7240, France

## 

Primary ciliary dyskinesia (PCD) is a rare congenital respiratory disorder characterized by abnormal ciliary motility leading to chronic airway infections. Qualitative evaluation of ciliary beating based on digital high-speed videomicroscopy (DHSV) analysis has been proposed to screen patients with suspected PCD. Our assumption was that quantitative analysis of ciliary beating would allow more precise identification of ciliary beat pattern abnormalities. Nasal nitric oxide measurement, nasal brushings and biopsies were performed prospectively in 26 consecutive patients with suspected PCD. In combination with qualitative analysis, 12 quantitative parameters of ciliary beat pattern were determined on DHSV recordings of beating ciliated edges. The “gold standard” (combination of ciliary ultrastructural abnormalities with nasal nitric oxide levels) excluded PCD in 7 patients (non-PCD patients), confirmed PCD in 10 patients (PCD patients) and was inconclusive in 9 patients. Among the 12 parameters, the distance traveled by the cilium tip and the area swept by the cilium, weighted by the percentage of beating ciliated edges both presented 96% sensitivity and specificity.In the PCD patients, quantitative analysis was concordant with the “gold standard”, while the qualitative evaluation was discordant with the “gold standard” in 3/10 cases. Among the patients with an inconclusive “gold standard”, the use of quantitative parameters supported PCD diagnosis in 4/9 patients and PCD exclusion in 2/9 patients. This study suggests that quantitative parameters provide a more precise description of ciliary beat pattern than qualitative evaluation, especially when ciliary beat pattern is moderately impaired (up to 40 % of patients with suspected PCD).

